# Fibromuscular Dysplasia Presenting as a Renal Infarct

**DOI:** 10.7759/cureus.2326

**Published:** 2018-03-14

**Authors:** Sidra Khalid, Jad H Daw, Ashoka Nautiyal, Hamed Daw

**Affiliations:** 1 Internal Medicine Residency, Fairview Hospital, Cleveland Clinic, Cleveland, USA; 2 School of Arts and Sciences, Case Western Reserve University, Cleveland, USA; 3 Department of Cardiology, Fairview Hospital, Cleveland Clinic, Cleveland, USA; 4 Department of Hematology and Oncology, Fairview Hospital, Cleveland Clinic, Cleveland, USA

**Keywords:** fibromuscular dysplasia, renal infarct, anticoagulation, hypertension

## Abstract

Fibromuscular dysplasia (FMD) is a condition caused by an abnormal development or growth of cells in the arterial walls in the body. We present a case of a 49-year-old male who came in with a sudden onset of severe left-sided abdominal pain. Computed tomography (CT) scan of the abdomen was suggestive of a left renal infarct. He underwent renal angiography that showed FMD and a clot located in the anterior branch of the left renal artery. The patient was then treated with apixaban for the clot and amlodipine for the associated hypertension. Our case will highlight the importance of recognizing renal infarction as an initial presentation of FMD.

## Introduction

Fibromuscular dysplasia (FMD) is a rare disease that is non-atherosclerotic and noninflammatory. It typically affects the small and medium sized arteries. It can cause stenosis, aneurysms, or tears in almost all of the arterial beds. FMD can rarely present as a renal infarct along with hypertension. It is diagnosed with digital subtraction angiography. Management of FMD includes anticoagulation, endovascular therapy, or in some cases open surgery. Patients with hypertension or renal impairment can be treated with percutaneous transluminal angioplasty (PTA) without a stent [1]. FMD is rarely diagnosed with renal infarction; hence our case will delineate the management of FMD presenting with renal infarction.

## Case presentation

**Figure 1:**
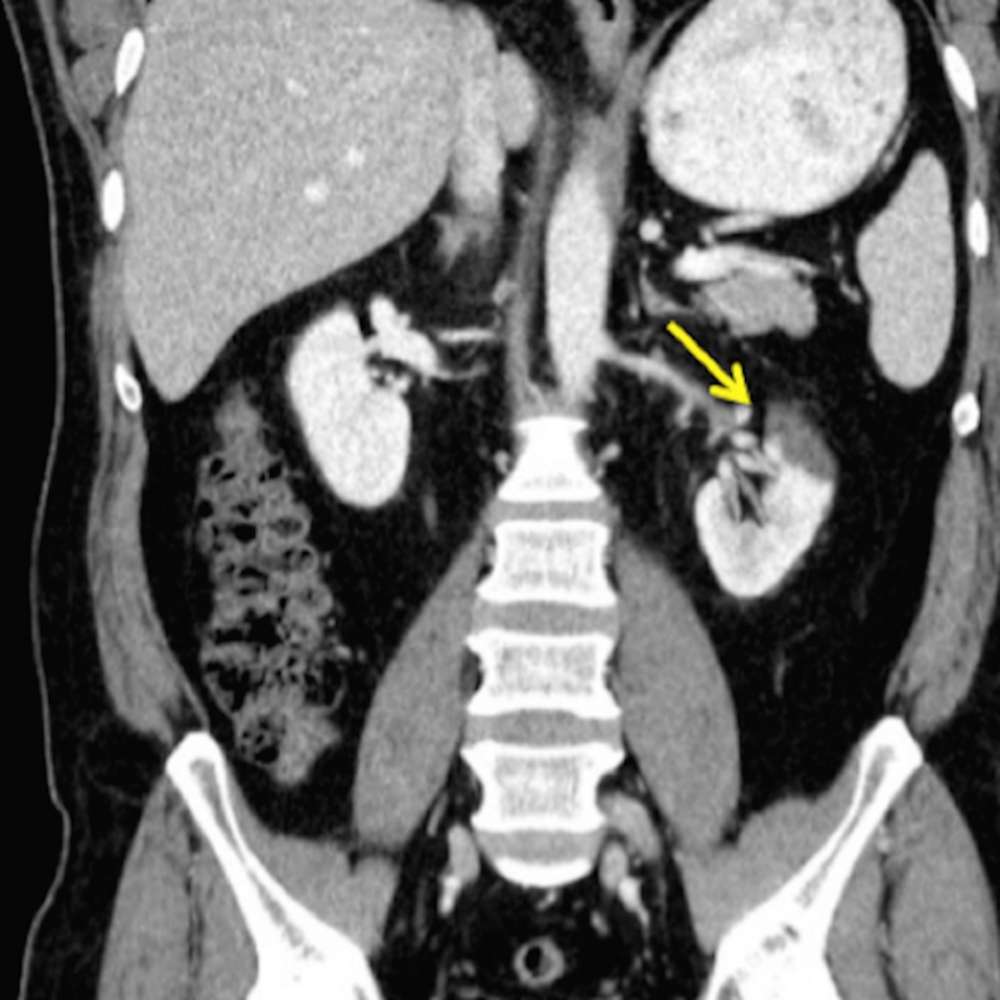
CT scan of abdomen and pelvis - poor opacification of the upper segment of the left kidney, suggestive of infarction (arrow) CT - computed tomography

**Figure 2:**
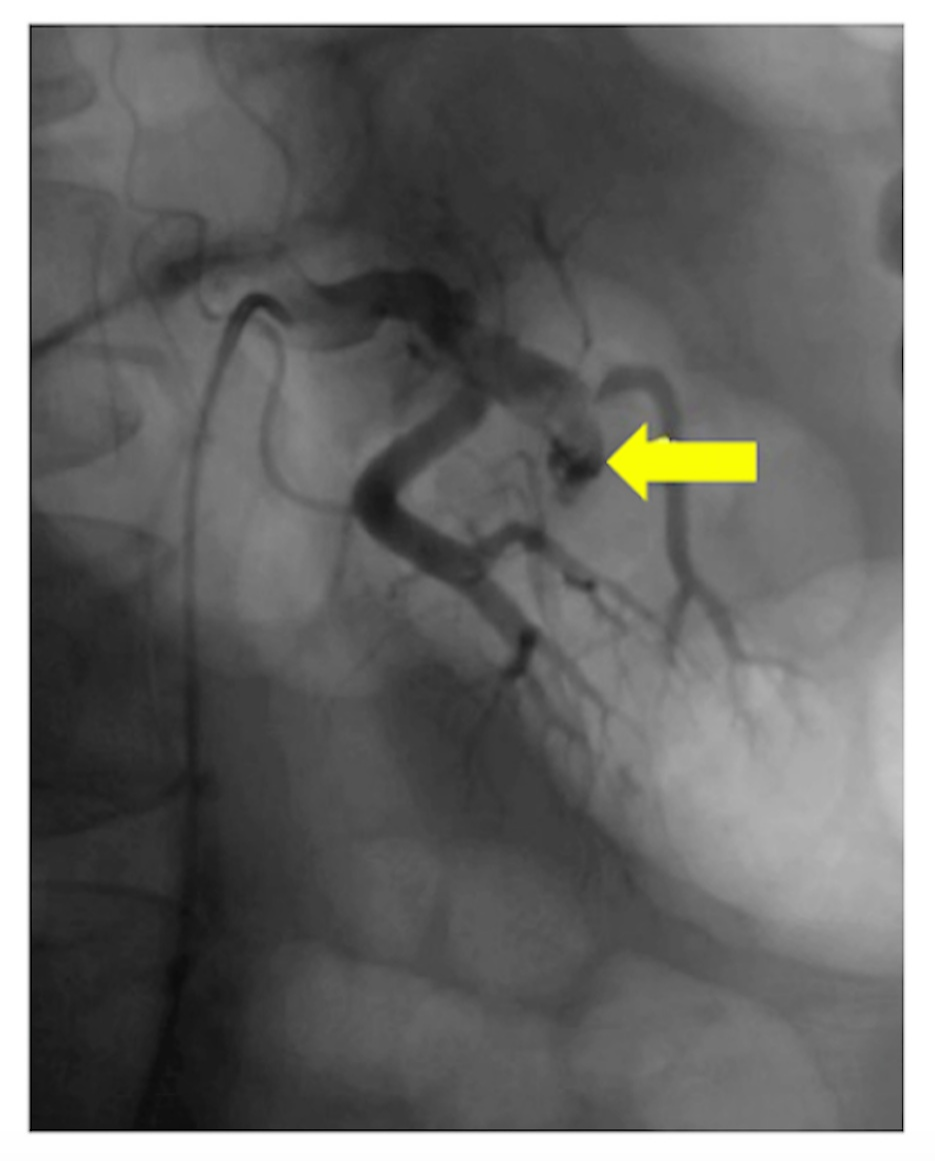
Renal artery angiography - FMD and clot in anterior branch of left renal artery (arrow) FMD - Fibromuscular dysplasia

A 49-year-old male presented with a sudden onset of severe, left-sided abdominal pain radiating to the groin for one day. He is a nonsmoker with a nonsignificant past medical and surgical history. His family history is contributory for factor V Leiden mutation in his brother and factor XII deficiency in his half-sister. On examination, his vital signs were afebrile, with a blood pressure (BP) of 150/90 mmHg, pulse 55/min, respiratory rate (RR) 22/min, and an unremarkable physical examination except for generalized abdominal pain on palpation. A computed tomography (CT) scan of the abdomen and pelvis showed poor opacification of the upper and interpolar segments of the left kidney, which were concerning for a renal infarct (Figure 1). To rule out an embolic source, transthoracic echocardiography was performed, which showed no evidence of a thrombus in the heart. To further delineate the underlying pathology and for revascularization, a renal artery angiography was planned. The angiography showed FMD with a clot in the anterior branch of the left renal artery (Figure 2). The patient was started on apixaban for the clot and amlodipine for hypertension. In the outpatient setting, renal artery duplex showed 0-59% stenosis of the left renal artery. Carotid and abdominal visceral arterial ultrasounds were unremarkable. Apixaban was discontinued after one month of therapy as the patient was asymptomatic with a BP of 120/82 mmHg. In the following six months, he underwent repeat imaging with a CT angiography (CTA) of the abdominal vasculature, which showed resolution of the clot and a beading pattern suggestive of FMD.

## Discussion

FMD is a disease that can present as stenosis or aneurysms of arteries. FMD typically affects the renal and internal carotid arteries but can appear in any arterial bed. It most commonly affects females, especially in the age range of 15-50 years [1]. Typically, patients with FMD are asymptomatic for many years. Often times, the diagnosis is made incidentally. Disease manifestations can be a result of ischemia related to stenosis, spontaneous dissection of arteries, rupture of aneurysms, or embolization of intravascular thrombi from aneurysmal segments. FMD presents as renal infarction with an incidence of 5.7% [2]. Hence, if a patient who presents with abdominal pain is found to have a renal infarct, FMD should be considered in the differential diagnosis. Other differentials of a renal infarct would include thromboembolism from cardiac arrhythmia or structural disease. Other potential causes include heart tumors or infective endocarditis, as well as hypercoagulability, autoimmune disorders, sickle cell disease, and aortic interventions [3].

Patients with renal infarction most commonly present with flank pain, but fever, vomiting, and oliguria can also be seen. Hematuria can arise in about half of the cases, and lactate dehydrogenase tends to be elevated. The diagnosis of FMD can be made with digital subtraction angiography, CTA, magnetic resonance angiography (MRA), or duplex ultrasonography. Angiography of the vessels can either present as a beaded or focal stenosis appearance [4].

When FMD is associated with renal infarction it can present with secondary hypertension. About 10% of patients who present with hypertension show FMD [3]. The main goal of managing patients with renal FMD is to control hypertension. Typically, drug therapy is an effective way to combat hypertension. Treatment is usually based on the presence of renal artery stenosis or renal hypoperfusion. The primary mechanism of hypertension in patients with unilateral renovascular FMD is the activation of renin-angiotensin-aldosterone (RAAS) system. The contralateral kidney maintains adequate natriureses, which means it avoids aldosterone-mediated sodium and fluid retention. In bilateral disease, RAAS blockers could lead to perturbation of the renal hemodynamics; therefore, the renal function needs to be monitored thoroughly. Diuretics can be a second-line therapy in unilateral disease or potentially as first-line in bilateral cases. Patients with a more persistent hypertension, resistance to treatment, or deterioration in renal function from ischemic nephropathy should be treated with revascularization. In most cases of renal arterial FMD, percutaneous transluminal angioplasty is preferred to surgery. Surgery is typically avoided except in the instance of macroaneurysms or complex lesions [4,5].

There is little data with regard to the natural history of renal infarction. One way to treat renal infarction is with anticoagulation. In a study of 438 Korean patients with renal infarction, anticoagulation proved to be effective in most patients and showed a low recurrence rate [6]. Therefore, management of FMD with renal infarction and hypertension depends on blood pressure control, anticoagulation, or revascularization with PTA or surgery. Also, since in 65% of cases, FMD involves other vertebral arteries, additional vessel imaging is necessary [2].

## Conclusions

Our case shows that FMD could present as a renal infarction with hypertension. Therefore, prompt diagnosis of the condition is needed to effectively manage patients and screen for FMD in other parts of the body.
